# Self-Administrated Elder Abuse Screening Tool for Older Adults With Visual and Hearing Disabilities

**DOI:** 10.1093/geroni/igab046.581

**Published:** 2021-12-17

**Authors:** Sarah Swierenga, Fuad Abujarad, Jennifer Ismirle, Chelsea Edwards

**Affiliations:** 1 Michigan State University, Michigan State University, Michigan, United States; 2 Yale University, New Haven, Connecticut, United States

## Abstract

Older adults age 60+ with disabilities are at greater risk of elder abuse compared to those without disabilities. We will describe results from our study to evaluate the usability and feasibility of the VOICES tablet-based elder abuse screening tool with older adults who have visual and hearing disabilities. VOICES is a digital health tool that screens, educates, and motivates older adults to self-report elder abuse. The VOICES tool has been developed and tested to be used with older adults without disabilities. We conducted a usability study with (n=14) older adults who were blind, had low vision, or were hard of hearing. Our evaluation method included both quantitative and qualitative measures to evaluate the ease of use and usefulness of the VOICES tool. Usability was measured as the percentage of tasks completed successfully, the average time to perform a task and the issues observed during performance of the tasks. Usability satisfaction was measured by written or verbal feedback on the questionnaires, and verbal comments from each session. Six participants completed the tasks successfully on their own; seven participants (mostly blind participants) completed the tasks with some intervention or help from the moderator. The majority of participants had System Usability Scale (SUS) scores 80 or above. Of all the participants, twelve (92%) stated that they would recommend the VOICES tool to others. Our findings generated universal considerations for more inclusive digital health interventions that accounts for the needs, wants and limitations for older adults with disabilities.

